# A Bibliometric Analysis of the Top 100 Papers on Gluteal Augmentation

**DOI:** 10.1093/asjof/ojae053

**Published:** 2024-07-08

**Authors:** Kian Daneshi, Hamid Reza Khademi Mansour, Niels Pacheco-Barrios, Ayobami Asaju, Mauricio Pérez Pachon, Alfredo Hoyos, Ankur Khajuria

## Abstract

**Background:**

Gluteoplasty or gluteal augmentation is a popular cosmetic procedure that is used to improve the volume, shape, and contour of the buttocks.

**Objectives:**

This bibliometric analysis aims to characterize emerging research trends and to assess the methodological quality of the highest impact gluteoplasty research.

**Methods:**

The 100 most-cited publications in gluteoplasty were identified on Web of Science, across all available journal years (from Inception to August 2023). Study details, including the citation count, main content focus, and outcome measures, were extracted and tabulated from each publication. Oxford Centre for Evidence Based Medicine level of evidence (LOE) of each study was assessed.

**Results:**

The 100 most-cited publications regarding gluteoplasty were cited by a total of 2375 publications. Citations per publication ranged from 5 to 176 (mean 23.75 ± 25.86), with the highest-cited study being authored by Simonacci, discussing autologous fat grafting (*n* = 176). Most publications were LOE 5 (*n* = 55), representative of the large number of case series and reports. The number of publications for LOE 1, 2, 3, and 4 was 1, 9, 13, and 22, respectively. The main content focus was “surgical technique” in 38 publications, followed by “outcomes” (*n* = 34) and “risk factors/prognosis” (*n* = 10). Patient-reported outcome measures (PROMs) were used in 20 publications, and 33 publications reported aesthetic outcome measures.

**Conclusions:**

This analysis demonstrates a need for improvement in research methodologies regarding gluteoplasty research. This advancement would be facilitated by robust, high-quality research through randomized control trials and multicenter studies, as well as the further development of validated PROMs for gluteoplasty.

**Level of Evidence: 2:**

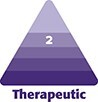

The popularity of gluteal augmentation has increased significantly in recent years especially in south and central America.^1^ The first description of this procedure was documented by Bartels et al in 1969 to correct buttock asymmetry by implanting breast silicone but it has now been used increasingly for aesthetic correction.^2,3^ The American Society for Aesthetic Plastic Surgery reported a 280% increase in the annual number of gluteal augmentations performed in the United States of America in 2015 compared with 2011.^4^ Within 2022 alone, >37,000 patients underwent buttock enhancement.^5^ This is partly due to the increased recognition of a “beautiful buttock” which contributes to beauty and attraction in most cultures as well as increasing awareness of social media beauty.^6,7^

Despite cultural and geographical differences in the understanding of ideal buttocks, Mendieta argued that buttock can be classified into 4 different shapes including A shape, V shape, round shape, and square shape. The author suggested that the A shape represents the most ideal shape with equal volume on all 4 sides of the buttock providing a more youthful look.^7^ Other assessment methods have been utilized to aid in surgical planning and define the ideal buttock shape, including the golden waist-to-hip ratio.^8,9^ Despite so, beauty remained subjective to the standards of the patient, and no standardized system has yet been implemented.

Surgeons have utilized different techniques to achieve gluteal augmentation. These include autologous fat transfer/grafting (gluteal lipoinjection), prosthesis implantation, autologous tissue flaps, hyaluronic acid gel injection, and combined surgical techniques.^10^ Nonetheless, autologous fat transfer and prosthesis implantation have remained as the commoner techniques preferred by surgeons, due to the low complication rate associated with autologous fat grafting and high satisfaction rate of prosthesis implants. Despite their advantages, both techniques have faced scrutiny due to their limitations, which include fatal complications such as fat embolism associated with autologous fat grafting, and high complication rates associated with implants.^6,10-12^ Autologous fat transfer has come under increased scrutiny due to its complication profile, which includes fat embolism and associated mortality; there have been 25 confirmed pulmonary fat embolism–related deaths in the United States over a 5.75-year time frame.^11,13-15^ The British Association of Aesthetic Plastic Surgeons recommended to ban the procedure in 2021. This has been lifted following safety regulations being brought in with the use of ultrasound-guided fat grafting.^16^

There has been a growing number of publications about gluteal augmentation from 1999 to 2021 as shown by a bibliometric analysis by Dai et al.^17^ Bibliometrics, the quantitative analysis of publications, especially scholarly literature, produces gainful insight into research trends and impact. Our study aims to provide a qualitative and quantitative examination of the existing articles on gluteal augmentation, including autologous fat transfer, to evaluate the current research trends and past research topics and to provide a prediction of future research objectives.

## METHODS

A literature review was performed to identify the 100 most highly cited publications on gluteoplasty. All journal publications available on the online database, Web of Science (Clarivate Analytics, Philadelphia, PA), were searched using the search terms: “gluteal fat grafting” OR “gluteal augmentation” OR “brazilian butt lift” OR “gluteal lipoinjection” OR “augmentation gluteoplasty” OR “buttock augmentation” as a “topic” on August 29, 2023. The timespan covered all available years (1973-2023). The inclusion criteria were journal publications from this search strategy. The exclusion criteria were publications not in English, papers not focused on gluteoplasty, animal studies, other surgical procedure, and duplicate publications. The level of evidence (LOE) was assessed as per the Oxford Centre for Evidence Based Medicine (OCEBM) system (2011).^18^

**Figure 1.**
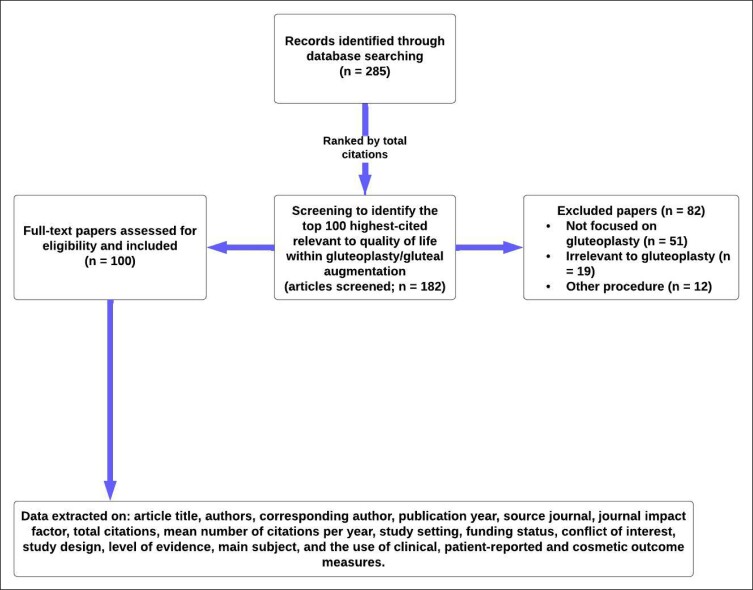
Summary flow chart of methodology. Screening and eligibility allowed the extraction of the 100 most-cited papers.

The search yielded a total of 285 journal publications, which were subsequently ranked based on the number of times cited. Publications with an equal number of citations were separated using the mean number of citations per year. To ensure that the publications were directly relevant to gluteoplasty, 2 reviewers (N.P.-B. and A.A.) independently screened titles and abstracts until 100 journal publications were included. Discrepancies were resolved by consensus discussion with the senior author (A.K.), with any remaining inconsistencies were ratified by review of the publication's full text. A total of 182 journal publications were screened to provide the most frequent 100 publications for inclusion. Reasons for exclusion of the other publications are specified in Figure 1.

Data were extracted from full-text publications in a standardized online computer spreadsheet (Google Sheets: Google LLC, Mountain View, CA). Data extraction included publication title, author list, corresponding author list, publication year, source journal, total citations, mean number of citations per year, geographical study setting, funding source, study design, LOE, main subject/content focus, declaration of conflict of interest (CoI), and the use of validated clinical, cosmetic, and patient-reported outcome measures (PROMs).

## RESULTS

### Citation Analysis

The 100 most-cited publications regarding gluteoplasty were cited by 2375 publications. The number of citations accumulated per paper ranged from 5 to 176. Publications were cited with a mean of 23.75 times ± 25.86. The mean number of citations per publication per year ranged from 0.44 to 25.14 (mean 2.96; Table 1). The top 100 studies from Table 1 have been referenced.^3,11,13,14,19-112^

**Table 1.** One Hundred Most-Cited Publications Regarding Gluteal Augmentation

Rank	Authors	Corresponding author	Total citations	Mean citations per year
1	Simonacci, F et al	Simonacci, F	176	25.14
2	Mofid, MM et al	Mofid, MM	134	19.14
3	Cárdenas-Camarena, L et al	Cárdenas-Camarena, L	105	11.67
4	Sinno, S et al	Wall, S	81	10.13
5	Gonzalez, R	Gonzalez, R	66	3.3
6	Murillo, W	Murillo, W	66	3.3
7	Oranges, CM et al	Oranges, CM	58	8.29
8	Conde-Green, A et al	Conde-Green, A	52	6.5
9	Mofid, MM et al	Mofid, MM	52	4.73
10	Villanueva, NL et al	Rohrich, RJ	51	8.5
11	Wolf, GA et al	Gallego, G	46	2.56
12	Del Vecchio, DA et al	Rohrich, RJ	42	7
13	de Pedroza, LV	de Pedroza, LV	42	1.75
14	Colwell, AS and Borud, LJ	Borud, LJ	40	2.35
15	Toledo, L	Toledo, L	38	4.22
16	Guisantes, E et al	Guisantes, E	34	2.83
17	Senderoff, D	Senderoff, D	34	2.62
18	Harrison, D and Selvaggi, G	Harrison, D	34	2
19	Nicareta, B et al	Pereira, LH	33	2.54
20	Astarita, DC et al	Astarita, DC	32	3.56
21	Serra, F et al	Serra, F	31	2.21
22	Cocke, WM and Ricketson, G	Cocke, WM	31	0.61
23	Centeno, RF and Young, VL	Centeno, RF	30	1.67
24	Mendieta, C	Mendieta, C	30	1.67
25	Vartanian, E et al	Macias, LH	29	4.83
26	Rosique, RG et al	Rosique, RG	28	2.8
27	Chopan, M et al	Katz, AJ	27	5.4
28	Abboud, MH et al	Dibo, SA	26	2.89
29	Serra, F et al	Serra, F	26	2.17
30	Wall, S et al	Wall, S	25	5
31	Centeno, RF	Centeno, RF	25	1.39
32	Cansancao, AL et al	Cansancao, AL	24	4
33	Sozer, SO et al	Sozer, SO	24	1.5
34	Hidalgo, J	Hidalgo, J	24	1.33
35	Rios, L and Gupta, V	Rios, L	23	5.75
36	Mendieta, CG and Sood, A	Sood, A	23	3.83
37	Kalaaji, A et al	Kalaaji, A	22	4.4
38	Ghavami, A and Villanueva, NL	Ghavami, A	22	3.67
39	Cardenas-Camarena, L et al	Cardenas-Camarena, L	22	2.75
40	Serra, F et al	Serra, F	22	2
41	Aboudib, JH et al	de Castro, CC	22	1.83
42	de la Pena, JA et al	de la Pena, JA	22	1.22
43	Willemsen, JCN et al	Lindenblatt, N	20	1.82
44	Flores-Lima, G et al	Flores-Lima, G	20	1.82
45	Rosique, RG and Rosique, MJF	Rosique, RG	19	3.17
46	Ramos-Gallardo, G et al	Ramos-Gallardo, G	19	3.17
47	Raposo-Amaral, CE et al	Raposo-Amaral, CE	19	01.06
48	Senderoff, D	Senderoff, D	18	2.25
49	de-Runz, A et al	de-Runz, A	18	2
50	Bayter-Marin, JE et al	Cardenas-Camarena, L	17	2.83
51	Singh, M et al	Talbot, SG	17	2.13
52	Del Vecchio, DA and Rohrich, RJ	Del Vecchio, DA	14	3.5
53	Pena, W et al	Cardenas-Camarena, L	14	2.8
54	Frank, K et al	Cotofana, S	14	2.8
55	Alvarez-Alvarez, FA et al	Alvarez-Alvarez, FA	14	2.8
56	Wang, G et al	Cao, WG	14	1.27
57	Asserson, DB et al	Asserson, DB	13	2.6
58	Mendieta, C and Stuzin, JM	Stuzin, JM	13	2.17
59	Salcedo, JAD et al	Salcedo, JAD	13	0.72
60	Serra, F et al	Serra, F	12	1.33
61	Cansancao, AL et al	Cansancao, AL	11	2.2
62	Menardias, B et al	Bertheuil, N	11	1.83
63	Andrade, GA et al	Coltro, PS	11	1.57
64	Hunstad, JP and Repta, R	Hunstad, JP	11	0.73
65	Del Vecchio, DA and Kenkel, JM	Del Vecchio, DA	10	5
66	Ordenana, C et al	Zins, JE	10	2.5
67	O'Neill, RC et al	Winocour, SJ	10	2.5
68	Shah, B	Shah, B	10	1.67
69	Levan, P and Habre, SB	Levan, P	10	1.43
70	Moscatiello, F et al	Moscatiello, F	10	0.71
71	Le Louarn, C and Pascal, JF	Le Louarn, C	10	0.63
72	Durairaj, KK et al	Durairaj, KK	9	2.25
73	Cardenas-Camarena, L and Duran, H	Cardenas-Camarena, L	9	1.5
74	Godoy, PM and Munhoz, AM	Godoy, PM	9	1.5
75	Swanson, E	Swanson, E	9	1.13
76	Paul, S et al	Paul, S	9	1
77	O'Neill, RC et al	Winocour, SJ	8	2.67
78	Che, DH and Xiao, ZB	Xiao, ZB	8	2
79	Everett, M et al	Everett, M	8	1.33
80	Nasseri, E	Nasseri, E	8	1
81	Jaimovich, CA et al	Almeida, AWR	8	0.57
82	Pazmino, P and Garcia, O	Pazmino, P	7	3.5
83	Cardenas-Camarena, L et al	Cardenas-Camarena, L	7	1.75
84	Pane, TA	Pane, TA	7	1.4
85	Chia, CT et al	Chia, CT	7	1.17
86	Wall, S and Del Vecchio, DA	Wall, S	7	1
87	Chang, H et al	Heo, C	7	0.44
88	Raposo-Amaral, CE et al	Raposo-Amaral, CE	7	0.44
89	Leyva, A et al	Leyva, A	6	1
90	Gonzalez, R and Gonzalez, R	Gonzalez, R	6	1
91	Vasilakis, V et al	Vasilakis, V	6	1
92	Whitfield, RM et al	Whitfield, RM	6	0.86
93	Muresan, C et al	Shureih, SF	6	0.6
94	Lourenço, LM et al	Lourenço, LM	5	2.5
95	Del Vecchio, DA et al	Del Vecchio, DA	5	1.67
96	Abboud, M et al	Abboud, M	5	1.67
97	Uz, I et al	Uz, I	5	1.25
98	Fadavi, D et al	Fadavi, D	5	1.25
99	Safran, T et al	Davison, PG	5	1.25
100	Singer, R	Singer, R	5	1

The most frequently cited publication (*n* = 176) was authored by Simonacci et al, and was published in 2017. The authors undertook a literature review and discussed the procedure, applications, and outcomes of autologous fat grafting (lipofilling).^19^ It was concluded that lipofilling can be applied to various plastic surgery procedures, namely gluteoplasty; however, its effectiveness could be limited by the amount of fatty tissue a patient possesses. Publication numbers for corresponding authors were recorded and the outcomes compared first. Cárdenas-Camarena had the largest number of authorships as corresponding author, at 5 each. This was followed by Serra (*n* = 4), with Wall and Del Vecchio both tied at 3 each (Table 1). Following corresponding authorships being calculated, active publications were counted next, with values for authors being calculated by the frequency of first authorships. Del Vecchio, Serra, and Cárdenas-Camarena were the authors with the most number of publications, with each having 4; however, Del Vecchio also had a publication as second author. This was followed by Mendieta at 3 and 6 authors all tied at 2 publications (Cansancao, Centeno, Gonzalez, Mofid, Wall, and Senderoff; Table 1).

**Figure 2.**
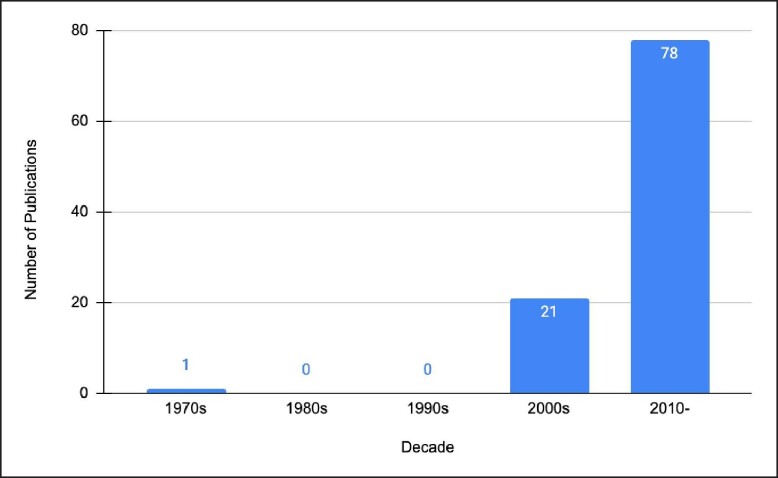
Decade analysis of the 100 most-cited publications in gluteal augmentation. These publications increase exponentially as decades progress.

Ninety-nine percent of the most frequently cited publications were published in the 2000s and 2010–, with there being 21 publications in the 2000s and 78 publications from 2010 to present day. The most frequently cited publication was published throughout this post-2010 time period (Figure 2). The United States of America was the country with the highest number of publications (*n* = 50), followed by Brazil (*n* = 16) then Mexico (*n* = 9; Table 2).

**Table 2.** Country Frequency in the 100 Most-Cited Gluteal Augmentation Publications

Rank	Country	No. of publications
1	USA	50
2	Brazil	16
3	Mexico	9
4	France	4
5	Colombia	3
6	Belgium	2
7	China	2
8	Spain	2
9	Canada	1
10	Dominican Republic	1
11	El Salvador	1
12	Italy	1
13	Netherland	1
14	Norway	1
15	Peru	1
16	South Korea	1
17	Switzerland	1
18	Turkey	1
19	UAE	1
20	UK	1

Six studies formally acknowledged the receipt of funding, a smaller proportion of funding (*n* = 2) was provided by various private entities such as Allergan (Dublin, Ireland) and Suneva Medical (San Diego, CA). Two publications stated the receival of a grant from a university- or academic-based research organizations, such as The Aesthetic Foundation (Garden Grove, CA). The remaining 2 funding sources included various charitable and private research corporations. Sixty-nine publications explicitly stated receipt of no funding, and the remaining 25 studies did not specify whether they received funding or not from external (governmental, industry, institutional, etc.) or internal (departmental, divisional levels of organizations) sources. Furthermore, 12 publications disclosed potential CoI with most authors stating that they were paid consultants, or the products being studied were provided for by a private corporation. A further 67 publications explicitly stated that there were no CoI to declare and the remaining 21 did not specify, if there were any CoIs or not.

### Prevalent Research Themes

The most-cited 100 publications regarding gluteoplasty derived from 18 journals. *Plastic and Reconstructive Surgery* (PRS) contributed the most publications (*n* = 31), followed by *Aesthetic Surgery Journal* (*n* = 26), with *Clinics in Plastic Surgery* next (*n* = 13), then *Aesthetic Plastic Surgery* (*n* = 10). The remaining journals contributed 20 publications between them (Table 3). Most of the publications selected for this analysis were from dedicated plastic surgery journals such as *Plastic and Reconstructive Surgery* and *Aesthetic Surgery Journal*.

**Table 3.** Journal Frequency in the 100 Most-Cited Publications

Rank	Source journal	No. of publications	Impact factor
1	*Plastic and Reconstructive Surgery*	31	3.6
2	*Aesthetic Surgery Journal*	26	2.9
3	*Clinics in Plastic Surgery*	13	2.3
4	*Aesthetic Plastic Surgery*	10	2.4
5	*Plastic and Reconstructive Surgery Global Open*	3	1.5
6	*Journal of Plastic Reconstructive and Aesthetic Surgery*	3	2.7
7	*Annals of Plastic Surgery*	2	1.5
8	*Dermatologic Surgery*	2	2.4
9	*Annals of Medicine and Surgery*	1	1.7
10	*Cureus: Journal of Medical Science*	1	1.2
11	*Dermatologic Therapy*	1	3.6
12	*European Journal of Plastic Surgery*	1	0.5
13	*Journal of Cosmetic Dermatology*	1	2.3
14	*Journal of Cutaneous Medicine and Surgery*	1	2.3
15	*Journal of Forensic Sciences*	1	1.6
16	*Journal of Investigative Surgery*	1	1.9
17	*Seminars in Plastic Surgery*	1	2.0
18	*Turkish Journal of Trauma and Emergency Surgery*	1	1.1

Ten publications on the list were published in journals not specifically related to plastic surgery which includes *Dermatologic Surgery*, *Annals of Medicine and Surgery*, *Cureus: Journal of Medical Science*, *Dermatologic Therapy*, *Journal of Cosmetic Dermatology*, *Journal of Cutaneous Medicine and Surgery*, *Journal of Forensic Sciences*, *Journal of Investigative Surgery*, and *Turkish Journal of Trauma and Emergency Surgery.* Among all the journals included, PRS and *Dermatologic Therapy* have the highest impact factor (*n* = 3.6).

### Methodological Quality

**Figure 3.**
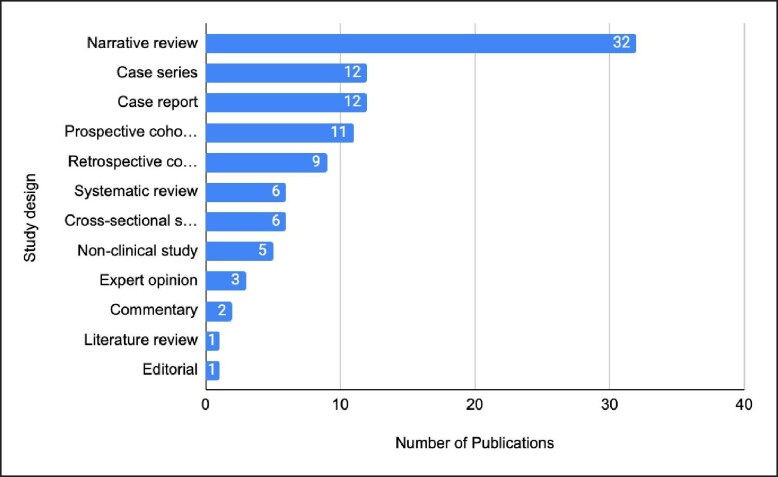
Study designs of the 100 most-cited publications on gluteal augmenation. Narrative reviews were the most popular study design.

Almost a quarter of the studies are cohort studies, with 9 having a retrospective model while 11 being prospective in nature. The most frequent study design utilized in this analysis was narrative reviews (*n* = 32), followed by case series and case reports tied (*n* = 12). Six systematic reviews were included; however, no randomized controlled trials (RCTs) are included in this study (Figure 3).

**Figure 4.**
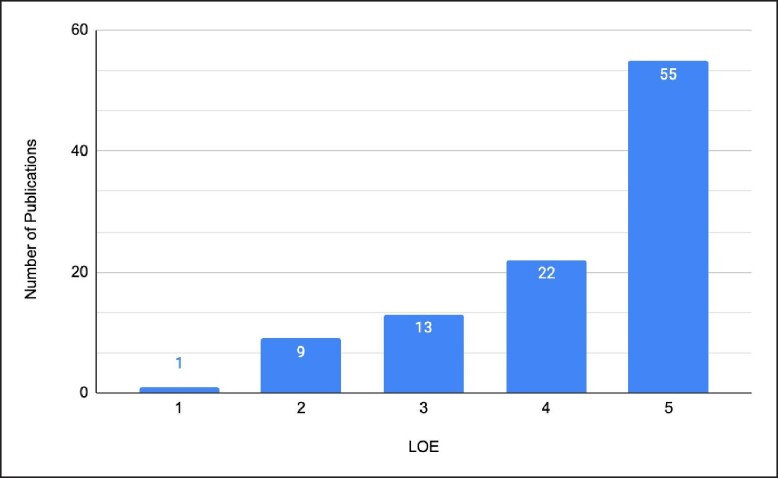
The LOE for the 100 most-cited gluteal augmentation publications. Most papers were LOE 5, with more of the remaining papers being a lower LOE than higher. LOE, level of evidence.

Over half of the publications on the list were assessed to be OCEBM LOE 5 (*n* = 55), this was represented mostly by case series and case reports. Twenty-two publications achieved LOE 4, while 13 publications achieved LOE 3. An additional 9 publications achieved LOE 2 and lastly 1 publication achieved LOE 1 (Figure 4). Upon observation of decade analysis, research output, in terms of the number of publications, greatly increased with each decade passing (1970s; *n* = 1, 1980s; *n* = 0, 1990s; *n* = 0, 2000s; *n* = 21 2010 to present; *n* = 78). Study designs of the 100 most-cited research studies on gluteal augmentation are presented in Figure 3.

Clinical outcomes were reported in 54 of the top 100 most highly cited publications. Outcome measures were categorized in studies. Twenty publications incorporated PROMs, namely patient satisfaction questionnaires, although a majority of these were unvalidated, generic scales. Thirty-three publications incorporated cosmetic outcome measures, including preoperative and postoperative photography, including the Global Aesthetic Improvement Scale and the Mendieta and Sood buttock ptosis classification.^51,113^

## DISCUSSION

This bibliometric analysis, conducted with careful adherence to the predefined methodology, reveals a surge in scholarly engagement and research output on gluteal grafting over the past 2 decades. This escalation reflects the evolution of aesthetic gluteal surgery, driven by innovations in surgical techniques and heightened prioritization of patient safety measures. The 2000s marked a significant turning point, highlighted by an exponential increase in scholarly publications—from none in the preceding decade to 2021. Moreover, an overwhelming 99% of the most-cited articles in gluteal grafting literature originate from this decade, underlining the period's critical role in this field's development. This finding coincides with the introduction of innovative fat grafting techniques and efforts to address safety concerns, particularly fat embolism syndrome—a significant risk associated with gluteal augmentation.^114^

The top 5 most-cited studies are a modest reflection of current research themes and present as a guide toward potential future literature trends and points toward the themes of our study. The main takeaways from Simonacci et al included that autologous fat grafting is safe for general applications; however, an inherent risk of complications exists when improper technique is applied in the buttocks.^19^ Both Mofid et al and Cardenas-Camarena et al highlighted the associated increased mortality rate in gluteal fat grafting, most likely due to gluteal blood vessel damage, causing microscopic and macroscopic fat embolism to occur.^20,21^ From Sinno's study, we can conclude that autologous fat grafting, when compared with silicone buttock implants, has lower complication rates overall.^11^ Lastly, in 2004 study, Gonzalez outlined the “XYZ method” of inserting implants, which was shown to be a safe method that produces very natural and long-lasting results, with very low rates of complication.^22^

Our study brings forth several important themes, namely the lack of validated PROMs applicable to gluteal augmentation and the optimization of safety—and by extension, the associated complications. Over a third of the studies’ main subject was “outcomes” (*n* = 34); a majority of these discussed the safety and efficacy of gluteal augmentation, where there were pitfalls and potential avenues of improvement. Nine studies explored complications as their main subject, where they mentioned all the various complications that can occur with gluteal augmentation and how their risk can be minimized. Our study demonstrates that there is a shortfall of objective evaluations in combination with patient satisfaction questionnaires, with the Mendieta and Sood buttock ptosis classification being the sole validated PROM utilized.^51^ This bibliometric analysis shows that although there are numerous studies exploring safety, more needs to be done to mitigate the risks associated with this only-increasingly popular procedure.

The pronounced contribution of research from the United States, Brazil, and Mexico (*n* = 50, 16, and 9 publications, respectively) reflects the greater demand for aesthetic gluteal procedures within these nations.^1^ It also reflects their pivotal role in pioneering the development of safer, more productive gluteal augmentation techniques. Such contributions have been instrumental in shaping international standards and practices in aesthetic surgery.^115^ Nonetheless, reliance on studies characterized by lower-level evidence (*n* = 55), such as case reports and expert opinions (LOE 5), reveals a void in the existing literature—the scarcity of rigorous, evidence-based research to inform and refine clinical practice and policy.^116^

A particularly optimistic discovery of this analysis is the growing focus on patient-reported outcomes (PROs) as a metric for assessing the success of gluteal augmentation surgeries. Within the realm of cosmetic plastic surgery, experienced surgeons acknowledge that many patients exist who have stellar results but are not satisfied with their results and the converse can also hold true—less than ideal results but the patient is extremely satisfied with their results; in some cases, this can be considered body dysmorphia, although it is context dependent.^117^ Consequently, it should be acknowledged that to move the field forward, quantitative evaluations must be combined with satisfaction-related outcome instruments.^118^ Ultimately providing plastic surgeons a means to determine whether the procedure was a success, failure, or somewhere in between. This paradigm shift toward patient-centric measures marks a stride toward acknowledging the centrality of patient satisfaction and overall quality of life as the most important benchmarks of aesthetic surgery's success. Integrating PROs into research has significantly enriched global comprehension of patient experiences, offering a well-rounded perspective on the efficacy of procedures beyond conventional clinical or cosmetic measures.^119^

The strength of this study lies in its exhaustive analysis of the worldwide research landscape on gluteal grafting, shedding light on prevailing trends, geographical variances, and thematic concentrations within the domain. This exploration aids in identifying specific areas for further inquiry, notably the need for high-caliber comparative studies to determine the safest and most effective gluteal augmentation techniques.

Nevertheless, this bibliometric analysis has inherent limitations. The primary concern among these is the potential inadequacy of citation counts as a measure of research impact, which might not fully represent the significance of more recent studies or those published in niche journals. Additionally, the focus on English literature may overlook relevant studies published in other languages, such as Portuguese or Spanish, possibly skewing the comprehension of global research trends.

Addressing these gaps necessitates future research efforts to prioritize the execution of RCTs and prospective cohort studies, which yield superior evidence on the safety, efficacy, and patient satisfaction rates associated with various gluteal augmentation techniques.^120^ Equally important is developing and validating standardized, PROMs tailored explicitly for gluteal augmentation patients. Such tools are already present and enable outcome comparisons across studies in facial and breast cosmetic surgery, enriching the evidence base that informs clinical practice and optimizes patient care in aesthetic surgery.^121,122^

## CONCLUSIONS

This extensive bibliometric analysis comprehensively examines the top 100 most highly cited publications regarding the gluteoplasty procedure and shows the evolution and trends in the field over the past 10 decades. Emerging research areas within this field include a multitude of refinements in safety and surgical technique to optimize for aesthetic outcomes and to mitigate complications. Improvements in the quality of gluteoplasty literature must be sought by active prioritization of the publication of methodologically robust studies with higher OCEBM LOE, such as well-designed RCTs or multicenter studies. Furthermore, the adoption of validated PROMs designed for gluteoplasty is centrally important for aligning patient satisfaction with clinical outcomes and providing high-quality evidence-based patient care.
